# Hypopituitarism and Other Endocrinopathies as a Consequence of Septo-Optic Dysplasia

**DOI:** 10.7759/cureus.82329

**Published:** 2025-04-15

**Authors:** Anjana Swami, Monish Sharma, Lindsey VanDyke

**Affiliations:** 1 Internal Medicine, Texas College of Osteopathic Medicine, Fort Worth, USA; 2 Radiology, Texas College of Osteopathic Medicine, Fort Worth, USA; 3 Endocrinology and Diabetes, Advanced Institute for Diabetes and Endocrinology, Mansfield, USA

**Keywords:** endocrinopathies, hormone therapy, optic nerve hypoplasia, pituitary hypoplasia, rare diseases

## Abstract

Septo-optic dysplasia (SOD) is a rare disorder that affects early brain development. It is characterized and diagnosed by the presence of at least two out of the following: optic nerve hypoplasia, agenesis of the septum pellucidum, and pituitary hypoplasia. Currently, the cause of the disease is largely thought to be sporadic and involves both genetic and environmental factors. We report the case of a 29-year-old female patient who was diagnosed with SOD after years of diagnostic investigation as well as the emergence of multiple endocrine pathologies related to her SOD. The patient is currently being treated with a variety of hormones to manage her endocrinopathies and is predicted to have a favorable prognosis.

## Introduction

Septo-optic dysplasia (SOD) is a rare disorder that is present at birth and affects one in 10,000 births but is often asymptomatic until early childhood. In addition to being a rare disorder, the prognosis is highly variable and depends on how many comorbidities are present [1,2]. Currently, the cause of the disease is largely thought to be sporadic in nature and involves both genetic and environmental factors [3]. We present a patient with SOD who presents with a more severe form of the disease. Much of the current literature is focused on the diagnosis and presentation of the disease in newborns and does not include how adults in stable conditions are being managed; this information is essential for the medical community, given that the incidence of SOD has increased in recent years [4].

## Case presentation

We present a 29-year-old female with a past medical history of panhypopituitarism secondary to SOD of the brain. The patient initially presented to the endocrinologist in 2022 for the management of her thyroid and pituitary glands. According to anecdotal birth history from her parents, the patient was born with no complications and was kept in the hospital for a few days for an abnormal Apgar score; she was eventually discharged with no definitive diagnosis or clues of underlying pathology. As the months progressed, her parents noticed that she was not meeting her motor and language developmental milestones. She was not able to crawl for six months and never made efforts to walk. Currently, the patient is in a wheelchair and is non-verbal. She communicates mainly with hand movements. 

Additionally, the patient was developing progressive vision loss, prompting further medical evaluation. For years, a diagnosis could not be determined, and she was managed symptomatically for her developmental, motor, and language delays. At age 3, she developed hypocortisolism, followed by diabetes insipidus (DI) at age 5. At age 7, from a combination of physical exam findings, hormone levels, and MRI, a formal diagnosis of SOD was reached. At her most recent follow-up visit, her physical exam included the presence of class II obesity, wheelchair restriction, thick neck size, +¼ patellar reflexes bilaterally, and poor insight and judgment into her clinical condition.

This patient's thyroid, vasopressin, and adrenal axes are affected; she is being managed for class II obesity complicated by cognitive impairment. Her midline brain abnormalities, such as agenesis of the septum pellucidum, are considered to be causing her inability to walk and her need for a wheelchair. She is currently in stable condition and continues to be monitored by her endocrinologist. There is regular monitoring of blood glucose levels, kidney function, liver function, thyroid function, and vitamin D levels (Table 1). Her doses for each of the medications are monitored closely and adjusted as needed (Table 2). Her vitals were also taken at every visit and reviewed for any abnormalities.

**Table 1:** Basic Metabolic Panel, Thyroid Function, and Vitamin D

Test	Value	Reference Range
Glucose, Non-fasting	84	70-100 mg/dL, Fasting
BUN	18	8-20 mg/dL
Creatinine	0.72	0.7-1.3 mg/dL
Sodium	143	136-145 meq/L
Potassium	3.8	3.5-5 meq/L
Chloride	107	96-106 meq/L
Carbon Dioxide	27	23-28 meq/L
Calcium	9.6	9-10.5 meq/L
T4, Free	1.5	0.9-2.4 ng/dL
Vitamin D	38	≥ 30 ng/mL

**Table 2:** Current Medication List With Regimen HS = at bedtime; PO AM = by mouth in the morning; PO PM = by mouth at bedtime; QAM = once a day in the morning

Medication	Regimen
Levothyroxine	137 mcg QAM
Desmopressin	0.2 mg tablet, three tabs PO AM, six tabs PO PM, and 1 mL injection HS
Hydrocortisone	10 mg AM, 7.5 mg PM

## Discussion

Much of the current literature focuses on the pathogenesis, pathophysiology, and diagnosis of SOD, especially in the newborn/pediatric population. In retrospective studies, it has been shown that infants diagnosed with SOD who also had visual and/or endocrine impairments commonly ended up with significant developmental delays and were considered to have severe SOD. This is in contrast to neonates who presented with isolated septal agenesis, who had relatively normal development [5].

Seeing that our patient presents with visual impairment, endocrine imbalances, and severe developmental delay, she can be categorized as having a severe form of SOD. Given her current, stable condition, this case may provide insight into how adults with even severe SOD can be managed given their multiple endocrinopathies. As the incidence of SOD has been rising in the past few years from one in 10,000 births, it is important that patients with SOD receive optimal care and management to keep the condition stable and to maximize their quality of life [3].

Since SOD only occurs in one in 10,000 births, it is not well known by physicians and usually diagnosed very late in childhood after multiple syndromes have already manifested. Currently, the cause of the disease is largely thought to be sporadic and involves both genetic and environmental factors. Warning signs associated with SOD include recurrent seizures, delayed development, growth restriction, and abnormal movements. The diagnosis can be confirmed with epileptic changes on EEG or optic nerve hypoplasia on retinal imaging. A definitive diagnosis is also confirmed with an MRI, which would show the absence of the septum pellucidum [6]. There are warning symptoms, such as persistent neonatal hypothermia, that can raise the suspicion of index for SOD and facilitate an earlier diagnosis [7]. In one study that detailed three infants with bilateral optic nerve dysplasia and SOD, ectopia or absence of the posterior pituitary bright spot was present and proved to be a sensitive MRI indicator of anterior pituitary dysfunction [8]. SOD should also be considered as a differential diagnosis when congenital mydriasis is present, which is another common initial sign of SOD [9].

The mainstay for the treatment of SOD is the management of the constellation of symptoms that accompany the disease. This includes eye exams by an ophthalmologist, hormone replacement, and early intervention or special education for any developmental delays [10]. Specifically, concerning hormone replacement therapy, there is no evidence to suggest patients with SOD should receive different hormonal regimens compared to patients without SOD who have the same deficiencies.

In terms of the patient presented in this case, she is being treated with desmopressin. This dosing is controlling her DI well and has almost eliminated any nocturia that the patient was having trouble with. The goal of effective treatment of DI includes fully eliminating nocturia and controlling daytime polyuria. It is important to control DI in these patients because there have been cases of sudden death in patients with SOD during stress events that have been linked to the dehydration caused by uncontrolled DI. Additionally, the patient with DI may not be able to attend to a thirst reflex secondary to her cognitive impairment. This can easily lead to frank dehydration and hypernatremia. Otherwise, someone with an intact thirst reflex and free access to unlimited water will be able to maintain appropriate sodium levels without any medical treatment. She is also being treated with hydrocortisone. This is because hypocortisolism in patients with SOD has also been fatal during a stress event [11]. Under her current dose, the patient's serum sodium and blood pressure are at goal. There is a stress dosing that applies to periods of acute illness and parenteral hydrocortisone to avoid adrenal crisis in an emergency. She is being treated with levothyroxine for her primary hypothyroidism. She was previously being treated with insulin for the management of her metabolic syndrome. Her elevated BMI can be attributed to her wheelchair restriction and her developmental delay, which prevent her from making typical lifestyle changes. 

There is currently a clinical trial focused on reducing obesity in patients with congenital thalamic injury, which includes patients with SOD. This trial tests the efficacy of Setmelanotide in suppressing hunger cues and improving weight reduction. Weight loss in these patients can improve their insulin resistance and make them less dependent on medication, among many other benefits. Patients such as the one presented in this case may benefit from this intervention, but more research needs to be done [12].

## Conclusions

SOD is a rare cause of impaired early brain development; however, it should always be on one’s differential list, especially if multiple endocrinopathies are arising throughout a child's life. Since the symptoms can be caught early and confirmed with imaging, there should be no delay in working up for SOD. Additionally, this case highlights the details and importance of a regimented treatment plan for patients with SOD, which is not illustrated in the current literature. Future studies should be conducted to investigate optimal treatment interventions depending on the stage of the disease.
